# The Burden of Likely Rubella Infection among Healthy Pregnant Women in Abakaliki, Ebonyi State, Nigeria

**DOI:** 10.1155/2022/5743106

**Published:** 2022-01-31

**Authors:** Uchechukwu Onyeukwu Ekuma, Ogbonnaya Ogbu, Angus Nnamdi Oli, Martin-Luther Oseni Okolo, Peter Anyigor Edeh, Hussein O. M. Al-Dahmoshi, Sousan Akrami, Morteza Saki

**Affiliations:** ^1^Department of Microbiology, Federal University of Science and Technology, Owerri, Imo State, Nigeria; ^2^Department of Applied Microbiology, Ebonyi State University, Abakaliki, Nigeria; ^3^Department of Pharmaceutical Microbiology and Biotechnology, Faculty of Pharmaceutical Sciences, Agulu, Nnamdi Azikiwe University, Awka, Nigeria; ^4^Department of Microbiology, Kogi State University Anyigba, Anyigba, Kogi State, Nigeria; ^5^Department of Biology, College of Science, University of Babylon, Babylon, Hilla City, Iraq; ^6^Department of Microbiology, Faculty of Medicine, Ahvaz Jundishapur University of Medical Sciences, Ahvaz, Iran; ^7^Student Research Committee, Ahvaz Jundishapur University of Medical Sciences, Ahvaz, Iran

## Abstract

The first 140 days of pregnancy are critical as regards rubella virus infection because of the likelihood of a poor pregnancy outcome. This study was undertaken to investigate the likelihood of exposure to poor pregnancy outcomes due to seroprevalence of rubella among selected pregnant women attending Mile Four Hospital, Abakaliki, Ebonyi State, Nigeria. The seroprevalence of rubella immunoglobulin M (IgM) antibodies was investigated among pregnant women. A total of 187 sera samples collected from the women were screened for rubella virus IgM antibody using the enzyme-linked immunosorbent assay (ELISA). The results obtained were analyzed using SPSS. The chi square test was performed at a *P* value of 0.05 significance and at a 95% confidence interval. Of the 187 pregnant women, 35 (18.72%) were positive for the rubella virus. Pregnant women within 26–30 years of age had the highest prevalence (26.15%), while those aged 35–40 years had the least prevalence. Married women had the highest prevalence (20.0%), followed by singles (16.67%) and widows (15.38%), while divorced pregnant women recorded the least prevalence (9.20%). Pregnant women with no formal education were more predisposed to rubella virus (22.22%) infection compared to their educated counterparts. Occupationally, full-time housewives had the highest prevalence (24.26%). The infection rates seemed to wane as pregnancy advanced. The first trimester had the highest prevalence (21.88%), followed by the second trimester (18.84%) and the third trimester (17.44%). Pregnant women living in urban areas had higher IgM seroprevalence (20.18%) than those in rural areas (16.67%). Furthermore, grand multigravidas were more infected (22.73%) than primigravidas (14.52%) and multigravidas (20.39%). The seroprevalence of rubella in this study was high, and it calls for general surveillance and mass immunization of children and females of childbearing age in the area to help reduce the incidence of congenital rubella syndrome.

## 1. Introduction

Rubella, also known as three-day measles, is an infectious disease caused by the rubella virus. The virus is a positive-sense single-stranded RNA virus that belongs to Togavirus family and has an envelope that originates from the host cell's plasma membrane [1, 2]. Although rubella is not as highly contagious as measles, both are vaccine-preventable acute viral diseases that affect males and females of all ages [3, 4]. Rubella without doubt remains a very important pathogen with global relevance, with approximately 105,000 cases of congenital rubella syndrome (CRS) occurring each year [5]. Although it is preventable through vaccination, the disease is one of the most infectious viral diseases known in human history with increasing morbidity and mortality in both mothers and fetuses [6]. It often results in lifelong immunity against reinfection [7, 8]. During pregnancy, when the rubella virus vertically infects the fetus from the mother, CRS occurs with the possibility of miscarriages and spontaneous abortions, cardiac disorders, cataract, deafness, cleft palate, autism, and sometimes fetal death [6, 7, 9]. The virus is transmitted through airborne droplets of infected people and is an acute, usually mild viral disease that commonly affects susceptible children and young adults worldwide [7, 8].

Rubella infections are usually asymptomatic with unreliable clinical diagnoses, and the diagnosis of acute infection in pregnant women most often relies on serological evidence. With this, immunologic tests such as the enzyme-linked immunosorbent assay (ELISA) and enzyme immunoassay (EIA) involving the detection of specific IgM and IgG antibodies are the most sensitive and prominent protocols for the identification of these infections for treatment [9].

With the inherent differences in cultural, economic, and medical history, the prevalence of rubella infection in pregnant women varies from country to country, and there is a need to study the status in different regions of every country. In Nigeria, pregnant women are not routinely screened for rubella. Screening to provide epidemiological data on the seroprevalence of rubella is therefore pertinent to create awareness and sensitize healthcare administrators and providers. There is a scarcity of information on the seroprevalence of rubella in Abakaliki, Ebonyi State, Nigeria. Hence, this study aimed to determine the seroprevalence of rubella IgM among pregnant women attending antenatal clinics to provide epidemiological data in this regard.

## 2. Methods

### 2.1. Study Sites and Population

The ethical approval for the current study was obtained from the Ethical Board of the Mile Four Hospital. Written informed consent form was provided to each of the subjects. The study site was the Mile Four Hospital (a Roman Catholic Mission-owned hospital) in Abakaliki, Ebonyi State, Nigeria. It was founded in 1946 for the management and care of leprosy and tuberculosis patients, but due to the scarcity of healthcare facilities in the state, it was upgraded to also take care of other health needs including antenatal care.

The study population consisted of all the pregnant women that attended antenatal care in the hospital from October 2014 to April 2015. The inclusion criteria include apparently healthy women who were clinically pregnant and aged between 15 and 50 years accessing antenatal care at the study site, while the exclusion criteria were non-pregnant women, pregnant women who did not consent to participate in the study, pregnant women who were not accessing an antenatal clinic at the study site, those outside the age group (<15 or >50 years), and pregnant women with a history of previous rubella vaccination.

### 2.2. Sample Collection and Processing

Consenting pregnant women were recruited consecutively after the purpose and procedure of the research had been fully explained to them. A pretested, validated questionnaire was provided to obtain demographic information. 5 ml of blood sample was collected aseptically by venipuncture from each consenting pregnant woman by a trained physician and centrifuged at 3000 rpm for 5 minutes. The sera were extracted with an Eppendorf pipette and stored at −20°C until IgM assay.

### 2.3. Sample Analysis

Rubella immunoglobulin M (IgM) antibody was determined in the sera of pregnant women using the quantitative rubella IgM specific enzyme-linked immunosorbent assay (ELISA) test kits (Microimmune Limited, UK). The samples were analyzed in accordance with the manufacturer's instructions. The mean value of the rubella IgM was calculated by dividing the mean absorbance values by the cutoff calibrator value. According to the used kit, samples with index values of >0.90 were considered positive while those with index value of ≤0.90 were considered negative.

### 2.4. Data Analysis

The data obtained were analyzed using the Statistical Package for the Social Sciences (SPSS) (Armonk, NY, USA) version 22.0 software while the chi square test was performed at a *P* value of 0.05 significance and at a 95% confidence interval.

## 3. Results

The overall prevalence of 18.72% was recorded for IgM seropositivity among the pregnant women in this study. Pregnant women within the age of 26–30 years recorded the highest prevalence of rubella IgM (26.15%), while zero prevalence was recorded by those ≥41 and ≤15 years old, respectively (Table 1). Married women recorded the highest prevalence of 20.00% while divorcees had the least prevalence of 9.10% (Table 2). Educationally, pregnant women with no formal education were more infected (22.22%) while those with primary education recorded the least prevalence of 13.51% (Table 2). Also, housewives recorded the highest prevalence of 24.36% while the traders had the least prevalence of 10.53% (Table 2).

Pregnant women in their first trimester had the highest burden with a prevalence of 21.88% while those in their third trimester recorded the least prevalence of 17.44% (Table 3). Pregnant women from urban areas recorded a higher prevalence of 20.18% than their rural counterparts (16.67%) (Table 3). Grand multigravidas recorded the highest rubella IgM seroprevalence of 22.73% while primigravidas had the least prevalence of 14.52% (Table 3).

## 4. Discussion

This study was carried out on a total of 187 pregnant women. They were assayed for rubella IgM antibody. The seropositivity of IgM for rubella virus was confirmed in the sera of 35 (18.72%) pregnant women that lends credence to earlier findings in several parts of the country [10–14]. This evidence should serve to sensitize healthcare planners, administrators, and providers that routine screening of pregnant women in Nigeria for rubella should be part of antenatal care. Our findings also supported the findings of Tamirat et al. [15] from Hawassa City, Southern Ethiopia, and Mirambo et al. [16] from Tanzania, all of which are developing countries. It has been shown that seroprevalence data are vital in the estimation of the global burden of CRS [17, 18]. The World Health Organization posited that IgM antibodies can be detected in serum even up to one year [19]. These women in whom IgM antibody were detected in their sera may likely be lacking IgG antibodies or protective immunity. Although in the current study, the seropositivity of IgM for rubella virus was below 20.00%, epidemiological studies always provide a good basis for future planning, including vaccination of high-risk populations such as pregnant women. Also, the results obtained by this study encouraged us to design another similar research for general population in Nigeria to evaluate their rubella seropositivity status.

This study showed the highest rubella IgM seroprevalence among pregnant women aged 26–30 years (26.15%) which was in accordance with the work of Ogbonnaya et al. [14] from Abia, Nigeria, and Okolo et al. [11] from Vom, Nigeria. Nevertheless, this finding was in contrast with the reports of Agbede et al. [20] from Ilorin, Koki et al. [10], from Kano, and Oyinloye et al. [12], from Borno State, Nigeria. Since women in this age group (26–30 years) are sexually active, they are more likely to be exposed to and infected by rubella virus [21]. However, older women (>40 years old) had the lowest rate of rubella IgM seropositivity. One possible reason is that with advancing age, the immune system shows less ability to produce appropriate antibody responses to antigens [22].

This study further revealed that married women were more infected (20.00%) than singles (16.67%), divorced (9.10%), or widows (15.38%). Although the mechanism involved in having higher rubella IgM seropositivity in married pregnant women than in single women is not known and more studies are needed, one possible reason may be due to the heterogeneity of the study population and the higher exposure of married women than single women because of having a sexual partner. This was in conformity with the findings of Ogbonnaya et al. [14] from Nigeria and Tamirat et al. [15] from Ethiopia, who detected rubella IgM antibodies in only married women.

The findings showed a higher burden of rubella infection among illiterates (22.22%) compared to women with primary (13.51%), secondary (20.73%), or tertiary education (18.67%). It is possible that this was due to unawareness of the rubella virus among the illiterate group. This was contrary to the findings of Gubio et al. [21] from Zaria, Nigeria, where there was no difference among the studied groups. This study further revealed that housewives had the highest rubella IgM seroprevalence (24.36%) compared to traders (10.53%), civil servants (14.63%), and farmers (20.00%), which was in agreement with the findings of Khan et al. [23] from Pakistan. Because housewives are likely to live in larger and more populous families and are in closer contact with others, the transmission of the rubella virus occurs more easily [23].

Women who were in their first trimester of pregnancy had the highest seroprevalence (21.88%). This was in agreement with the work of Gubio et al. [24] from Kaduna, Nigeria, and Tamirat et al. [15] from Ethiopia, but in contrast with that of Agbede et al. [20] from Ilorin, Nigeria, who reported a greater vertical transmission of rubella during the second trimester. Also, Oyinloye et al. [12] recorded the highest rubella seroprevalence among pregnant women in their third trimester. Rubella IgM seropositivity was higher in the first and second trimesters, possibly because in those trimesters, women were considerably younger than women in the third trimester. Because most young women are still susceptible to the rubella virus, being younger has been linked to an increased risk of acute rubella virus infection [25].

Finally, more infection was found among urban dwellers (20.18%) than in women from rural places (16.67%) that was in corroboration with the reports of Tamirat et al. [15] and Wondimeneh et al. [26] from Ethiopia. The reason for the higher rubella seropositivity of women living in urban areas is the higher population density in these areas and the increased chance of contact that may lead to infection in people without enough protective levels of rubella immunity [26].

Grand multigravidas had the highest prevalence (22.73%) compared to primigravidas (14.52%) and multigravidas (20.39%). This was contrary to the work of Agbede et al. [20] and Tamirat et al. [15], who observed the highest prevalence among primigravidas in Ilorin, Nigeria, and Ethiopia, respectively. The differences in the seroprevalence rates of rubella virus among pregnant women may be due to their cultural and sexual behaviors, geographical locations, socioeconomic class, ethnicity, or season of sample collection. The current study had some limitations due to the financial constraints. These include the lack of a rubella IgG assay and the lack of detection of live viral infection in pregnant women by quantitative polymerase chain reaction (qPCR).

## 5. Conclusions

This study showed the existence of rubella infection among healthy pregnant mothers in Abakaliki, Ebonyi State, Nigeria, suggesting a high risk of poor pregnancy outcome among the study population. Therefore, there is need for massive immunization, with the rubella vaccine, of women who are in their reproductive age in the area to help reduce the likelihood of congenital rubella syndrome and other possible poor pregnancy outcomes.

## Figures and Tables

**Table 1:** Age spread of rubella IgM antibody among pregnant women.

Age (years)	No. of people screened	No. of positive cases (%)	No. of negative cases (%)

≤15	2 (1.07)	0 (0.00)	2 (100.00)
16–20	17 (9.10)	4 (23.53)	13 (76.47)
21–25	43 (22.99)	9 (20.93)	34 (79.07)
26–30	65 (34.76)	17 (26.15)	48 (73.85)
31–35	36 (19.25)	4 (11.11)	32 (88.88)
36–40	19 (10.16)	1 (5.26)	18 (94.74)
≥41	5 (2.67)	0 (0.00)	5 (100.00)
Total	**187 (100.00)**	**35 (18.72)**	**152 (81.28)**

**Table 2:** Marital status, educational level, and occupational spread of the studied pregnant women.

Marital status	No. of people screened (%)	No. of positive cases (%)	No. of negative cases (%)

Married	145 (77.54)	29 (20.00)	116 (80.00)
Single	18 (9.63)	3 (16.67)	15 (83.33)
Divorced	11 (5.88)	1 (9.10)	10 (90.90)
Widow	13 (6.95)	2 (15.38)	11 (84.62)
Total	**187 (100.00)**	**35 (18.72)**	**152 (81.28)**
*Educational level*			
Primary	37 (19.79)	5 (13.51)	32 (86.49)
Secondary	82 (43.85)	17 (20.73)	65 (79.27)
Tertiary	59 (31.55)	11 (18.64)	48 (81.36)
None	09 (4.81)	2(22.22)	07 (77.77)
Total	**187 (100.00)**	**35 (18.72)**	**152 (81.28)**
*Occupation*			
Housewife	78 (41.71)	19 (24.36)	59 (75.64)
Trading	38 (20.32)	4 (10.53)	34 (89.47)
Civil servant	41 (21.93)	6 (14.63)	35 (85.37)
Farmer	30 (16.04)	6 (20.00)	24 (80.00)
Total	**187 (100.00)**	**35 (18.72)**	**152 (81.28)**

**Table 3:** Pregnancy stage, residence, and parity spread of the IgM antibody among study population.

Stages of pregnancy	No. of people screened	No. of positive cases (%)	No. of negative cases (%)

1^st^ trimester	32 (17.11)	7 (21.88)	25 (78.13)
2^nd^ trimester	69 (36.90)	13 (18.84)	56 (81.16)
3^rd^ trimester	86 (45.90)	15 (17.44)	71 (82.56)
Total	**187 (100.00)**	**35 (18.72)**	**152 (81.28)**
*Place of residence*			
Rural	78 (41.71)	13 (16.67)	65 (83.33)
Urban	109 (58.29)	22 (20.18)	87 (79.82)
Total	**187 (100.00)**	**35 (18.72)**	**152 (81.28)**
*Parity*			
Primigravida	62 (33.16)	9 (14.52)	53(85.48)
Multigravida	103 (55.08)	21 (20.39)	82 (79.61)
Grand multigravida	22 (11.76)	5 (22.73)	17 (77.27)
Total	**187 (100.00)**	**35 (18.72)**	**152 (81.28)**

## Data Availability

The data used to support the findings of this study are available from the corresponding authors upon request.
